# Combining mobile-health (mHealth) and artificial intelligence (AI) methods to avoid suicide attempts: the Smartcrises study protocol

**DOI:** 10.1186/s12888-019-2260-y

**Published:** 2019-09-07

**Authors:** Sofian Berrouiguet, María Luisa Barrigón, Jorge Lopez Castroman, Philippe Courtet, Antonio Artés-Rodríguez, Enrique Baca-García

**Affiliations:** 1 Department of Psychiatry and Emergency, Brest Medical University Hospital, Brest, France; 2 SPURBO EA 7479, Ubo, France; 3 CHRU Cavale Blanche University Hospital of Brest, Boulevard Tanguy Prigent, 29,609 Brest Cedex, Brest, France; 4 Inserm U1061, La Colombières Hospital, University of Montpellier, Montpellier, France; 5 Gregorio Marañón Health Research Institute, Madrid, Spain; 6 Department of Psychiatry, Autónoma University, 28040 Madrid, Spain; 7 Inserm U1061, CAC University Hospital of Nîmes, Nîmes, France; 8 Department of Psychiatry, Fundación Jiménez Díaz Hospital, 28040 Madrid, Spain

**Keywords:** Suicide, Data mining, Sensors, Smartphone, Wearables

## Abstract

**Background:**

The screening of digital footprint for clinical purposes relies on the capacity of wearable technologies to collect data and extract relevant information’s for patient management. Artificial intelligence (AI) techniques allow processing of real-time observational information and continuously learning from data to build understanding. We designed a system able to get clinical sense from digital footprints based on the smartphone’s native sensors and advanced machine learning and signal processing techniques in order to identify suicide risk.

**Method/design:**

The Smartcrisis study is a cross-national comparative study. The study goal is to determine the relationship between suicide risk and changes in sleep quality and disturbed appetite. Outpatients from the Hospital Fundación Jiménez Díaz Psychiatry Department (Madrid, Spain) and the University Hospital of Nimes (France) will be proposed to participate to the study. Two smartphone applications and a wearable armband will be used to capture the data. In the intervention group, a smartphone application (MEmind) will allow for the ecological momentary assessment (EMA) data capture related with sleep, appetite and suicide ideations.

**Discussion:**

Some concerns regarding data security might be raised. Our system complies with the highest level of security regarding patients’ data. Several important ethical considerations related to EMA method must also be considered. EMA methods entails a non-negligible time commitment on behalf of the participants. EMA rely on daily, or sometimes more frequent, Smartphone notifications. Furthermore, recording participants’ daily experiences in a continuous manner is an integral part of EMA. This approach may be significantly more than asking a participant to complete a retrospective questionnaire but also more accurate in terms of symptoms monitoring. Overall, we believe that Smartcrises could participate to a paradigm shift from the traditional identification of risks factors to personalized prevention strategies tailored to characteristics for each patient.

**Trial registration number:**

NCT03720730. Retrospectively registered.

## Background

### Suicide risk and monitoring

Over 800,000 people die because of suicide every year worldwide. For each suicide, there may have been more than 20 other attempted suicides. A previous attempt is the major predictor of death by suicide [1]. However, many other outcomes associated with suicidal behaviors should be considered in order to strength preventative strategies prevention. Environmental, Clinical, and genetic [2] risk factors have been intensively studied among suicide attempters in order to improve personalization and effectiveness of preventative programs [3]. Suicide risk assessment relies on standard follow-up outpatient visits in a psychiatric clinic. This assessment includes a mental status examination, a brief update on the history of the patient’s present illness (including a safety assessment of risk of self-harm, suicide). In order to develop a form of assessment that can more accurately track patients’ symptoms and risks between visits, we conducted several studies aiming to add ecological momentary assessment to traditional follow up strategies [4] [5]. Advances in sensors technology, and artificial intelligence methods (AI) also draw new perspectives for behavior ecological assessment [6]. Sleep disturbances and food intakes are not one of suicide risk factors usually screened in routine suicide risk monitoring [7].Yet this clinical information could play a central role in suicide risk identification and prediction.

### Sleep, eating disorders and suicide risk

Sleep loss and sleep disturbances are associated with an increased risk of suicidal behavior, including suicidal ideation, suicide attempt, and completed suicide [8]. This relationship is consistent across psychiatric diagnoses and age groups [9]. The increased risk of suicidal ideation and suicide attempts among patients with early or late awakening times was demonstrated in representative samples of Korean [10] and British [11] adolescents of both genders. Chui et al. observed in a pooled sample of adolescents a linear dose–response relationship between sleep duration and suicide plans [12]. Rossler et al. showed that the correlates between sleep disorders including short sleep duration and suicidality was also observed in adults [13]. In a 30-year longitudinal cohort study of adults people, authors showed that the more severe the sleep problems are, the more pronounced is suicidality, including suicide ideation and behaviours. Interestingly these patterns persist even if we control for socio-demographics and in particular for accompanying mood, anxiety, or substance-use disorders, [14]. Changes in appetite are closely connected with depressive symptomatology and suicidal behavior [15]. Appetite has already been used in the prediction of suicidal risk [16]. Regarding eating disorders, negative self-image variables have been associated with suicide attempt history in anorexia and suicide attempts completions in women with bulimia [17]. In fact, in a cohort study of eating disordered subjects, the risk for suicide attempt was 4.70 (95% CI 1.41–15.74) in patients with eating disorders compared to matched (age, sex, place of residence) general population controls and further increased to 11.30 (95% CI 6.90–18.50) in those patients with history of previous suicide attempt [18]. A recent study found that suicide attempters had lower body mass index (BMI) and waist circumference, as well as decreased levels of serum [19].

### Suicide risk management in the e-health era

The integration of smartphones and wearables into medical practice has heralded the electronic-health (e-health) era. E-health involves the integration of new technologies into routine clinical practice by increasing networking possibilities between patients and clinicians. Recent trials using mobile electronic devices have proven successful in real-world and real-time monitoring and have improved the assessment possibilities in a large panel of clinical settings [20]. The assessment of the patient’s dynamic relationships between events and disease course is enhanced by the development of momentary data collection strategies, such as experience sampling methods (ESM) and ecological momentary assessment (EMA) [21]. These approaches, which rely on delivering informative contents and self-administered questionnaires, reduce the recall bias as they are done in real time [22]. Electronic devices are also able to perform passive (or autonomous) data gathering, i.e., to extract information about the users without any effort on their part. Actigraphy, geolocation, and communication activity are usual features of current smartphones and may be indicators of the patient’s behavior if they are properly processed. Overall, an extensive panel of physical and mental conditions can be remotely monitored [23]. Smartphones can embody such sensors and provide EMA features relying on patient self-reports. Finally, the data digital footprints, which is the automatically accumulated by-products of technological devices, offer a promising opportunity for research and clinical decision making [24].

The collected data can be processed and transferred over the Internet to a remote clinical back-end server for further analysis, assessment, decision making and intervention. AI is a set of analysis methods which permits to extract relevant information from any dataset. AI uses data mining techniques to get sense for the data. a set of techniques that can be used to explore treatment and outcome questions in large clinical databases and help develop algorithms and guidelines for problems where controlled data are difficult to obtain. The data mining process includes several steps, including data capture, data selection, data processing, and machine learning. Abdullah et al. reported that combining self-reported data with data from several smartphone sensors and communication patterns resulted in reliable prediction of the Social Rhythm Metric, a clinically-validated marker of stability and rhythmicity for individuals with bipolar disorder [25] Voice features from phone calls can also be collected and analyzed during everyday life in naturalistic settings. For inferring rhythmicity, a key marker of wellbeing for individuals with bipolar disorder. Another platform proposed A method to predict schizophrenia based on anomaly detection was developed using Beiwe, a platform able to perform data mining on smartphone data [26]. EMA has been successfully used for real-time self-reporting of symptoms and behavior—for example, Husky et al. showed the utility and feasibility of using EMAs to study suicidal ideations [27].

Taking into account the strengths and pitfalls of existing suicide risk assessment methods, we have designed a system capable to combine EMA and continuous monitoring of patients using the smartphone’s and/or wearable’s sensors and data entry in order to monitor and predict suicide risk. Both poor sleep quality and disturbed appetite are clinical markers of depression, with a bidirectional relationship. Their changes could precede the increase of suicidal behavior as sleep and appetite are both influenced by mood state and regulated by the serotonergic system. Our hypothesis is that tracking sleep and appetite regularly through self-report could serve as a consistent and non-biased shortcut to assess mood state, its biological underpinnings and could potentially predict suicidal behavior.

### Objectives

The overall study goal is to determine the relationship between wish to die, and suicide attempt and changes in sleep quality and disturbed appetite using Smartphone and wearable technology based using ecological momentary assessment (EMA).

The principal objective of the study is to identify the correlation between quality of sleep monitored via the digital footprint and suicidal events including and suicide attempts in a cohort of suicidal patients.

Secondary objectives are:

- Establish the extent to which change in appetite is related to and suicide attempts.
- Clarify the timeline of the relationship between sleep disturbances and suicidal behavior.
- Determine the emotional impact of the use of the app during suicide related outcomes assessed.
- Develop personalized algorithms based on the EMA protocol and motor activity markers to assess the risk of suicide attempts.

## Method and design

### Study design and setting

This study is a pragmatic, prospective, multisite controlled trial with two parallel groups. The study setting is outpatient clinics and emergency departments at four clinical centers across two countries (Jimenez Diaz Foundation, Spain, University Hospital of Nimes, France).

### Participants

To be eligible for the study, patients must be 18 years or older, attend an outpatient clinic or emergency departments of participating clinical services and owning an android or IOS smartphone. Exclusion criteria are being less than 18 years old, refusal to participate to the study, and patients and in emergency situations where their state of health does not allow obtaining written consent. Patients who are unable or unwilling to provide informed and signed consent will be also excluded.

This study was built on the results of previous studies we conduced exploring the preferences of patient regarding eHealth based suicide assessment [28, 29]. Patients were involved in the design of this study in a preliminary step of the application conception which assed the feasibility and the burden of such approach in a sample of outpatients [30]. Patient were informed they could have access to the final result of the study during a routine medical visit, according to the ethic committee query.

### Intervention and control

After agreeing to participate, clinician will collect a signed consent for each patient. Patient will be assigned to intervention or control group weather they have a personal history of suicide attempt or not. Patient with a current suicidal behavior disorder (according to DSM-5), i.e. having attempted suicide in the current year, will be assigned to the intervention group.

**Fig. 1:**
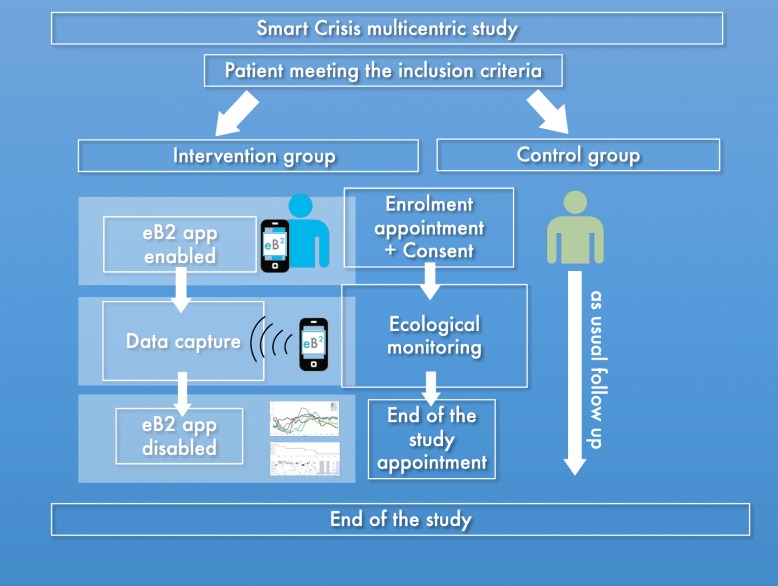
Patient flow in intervention group and control group

Patient with no history of suicide behavior will be assigned to the control group. Both intervention and control group will receive as usual follow up. monitoring visits. In the intervention group, each participant will be assessed with an EMA protocol using two smart phones apps. The first app will ask questions following a dynamic protocol (quality and duration of sleep, wish to live and wish to die, and questions regarding emotional status), the second app will record diurnal activity that is related with sleep quality using smartphone sensors. Additionally, a subsample of participants of the intervention group will be monitored with a wearable armband to assess sleep phases and physiological changes during sleep and EMA assessment. Control group will only receive as usual follow up procedure and monitoring procedure of the study protocol (see Fig. 1).

### Primary outcome

The primary outcomes are suicidal events assessed by Columbia-Suicide Severity Rating Scale (C-SSRS) in control and intervention group during the 6-month follow-up period.

### Secondary outcomes

Secondary outcomes are:

Evaluate appetite using the Council of Nutrition Appetite Questionnaire (CNAQ) score with EMA questions.

Sleep duration before and after a suicide attempt in intervention group during the 6-month follow-up period.

Electrodermal activity captured by waerables during EMA assessment in the intervention group.

Pooled EMA data Suicidal events and eating disorders in control and intervention group during the 6-month follow-up period.

### Digital collection, and data analysis

The “Smartcrises” platform is composed by two smartphone apps, which collect EMA data and smartphone sensors data, and a back-end server, which stores and processes them.

### EB2 app

The eB2 app collects data from inertial sensors, physical activity, phone calls and message logs, app usage, nearby Bluetooth and Wi-Fi connections, and location. Also, using Google Play Services, the app is able to access detailed activity information and nearby location data. The app was developed to run in background and the user only interacts with the app for the initial configuration. Moreover, it was designed with battery safe considerations like non-continuous recording schedule, automatic sleep/wake function, and it additionally notifies the operating system to relaunch itself when it was closed or stopped due to user’s actions or failures/reboots.

### Memind app

Memind is a smartphone application able to perform EMA. The MEmind app will assess wish to die via a self questionnaire and a computer assisted algorithm. The type and amount of food intake will be evaluated through the dynamic questions. Participants will be asked four static questions in the morning and evening while dynamic questions will be asked randomly at any one time-point during the day (at most 4 random questions from a pull of 32 questions distributed throughout the day). This application will offer questions on the quantity and quality of sleep, appetite and food intake, on the occurrence of social stress, evaluation of suicidal ideas and emotions. These questions are displayed according to a nonlinear dynamic model during participation to the study [31].

### Data collection and follow-up

#### Baseline assessment

The baseline enrolment questionnaire includes the patient’s basic demographic information including age, gender, marital status, employment status, country of birth, language spoken at home, level of education and whether they had a regular general practitioner.

Medical treatments at inclusion, as well as any changes in these treatments during follow-up, will be collected and analyzed as potential confounders. The treatments will be classified according to the classification established by the World Health Organization (ATC index). The entire lifetime diagnosis of mental disorders axis I and II according to DSM-5 will be based on existing clinical evaluation protocols at inclusion: Mini International Neuropsychiatric Interview 7.0.0. Participants will be evaluated for depressive symptomatology [32], to detect the possibility of a manic episode [33] and to evaluate their global functioning [34].

#### Outcomes measures

We will use complementary strategies to obtain the primary outcome measures and assess adverse events. Assessments will be carried out by trained clinicians (Psychologist, Psychiatrist or Psychiatrist Residents up to second year) at outpatient clinics and emergency departments.

Researchers will check the hospital attendance data especially the emergency records.

The assessment of suicidal behaviors will include at the inclusion and completion of follow-up:

- The Columbia Suicide Severity Rating Scale a scale that showed good sensitivity and specificity to identify the most at risk subjects [35].
- If a suicide attempt has occurred, the suicide intent scale [36] and the Risk Rescue Rating Scale.

#### Suicide and sleep monitoring

Evaluation of the principal objective will be monitored via in-person interviews (see baseline assessment subsection) and EMA self-questionnaires including wish to live, wish to die, suicidal ideation, and suicide attempt during the follow-up period. The Suicidal Status Form [37] the Perceived Social Support Questionnaire (PSSQ,) and the Interpersonal Needs Questionnaire [38] have been incorporated into pop-ups to our EMA app to increase the participation rate during inactive periods [31].

**Fig. 2:**
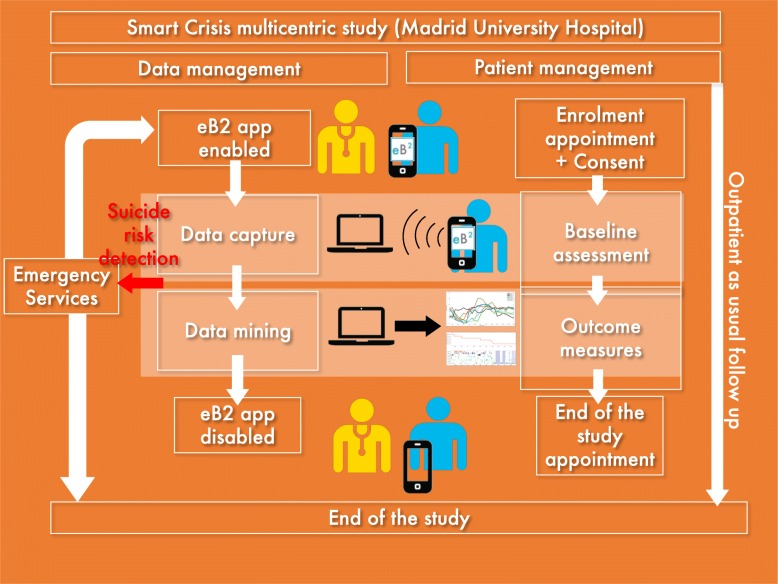
Patient flow in intervention group

The MEmind App will provide questions following a dynamic smartphone based assessment protocol exploring quality of sleep. The EB2 app will record diurnal activity that is related with sleep quality using smartphone sensors. EB2 will be validated with polysomnographic registers (see Fig. 2). EB2 is designed to collect information about the users’ physical and physiological activity level. This application has been developed for the Android mobile platform and works in combination with the arm Band. SmartPhone and the arm Band communicate via Bluetooth 4.0. This application obtains information from the user by accessing the built-in sensors in the host Android SmartPhone and the arm Band. Through the process of data logging the Sensor Logger APP records both the information from the SmartPhone and arm Band. This double recording of information by the Sensor Logger APP allows it to obtain enough information from inertial sensors to determine the user’s activity level during the recording process [39]. The heart rate signal and Galvanic skin response are also recorded to determine the emotional state of the user. In our study participants in the Smartcrisis group will be monitored with a wearable armband to assess sleep phases and physiological changes during sleep.

#### Food intake monitoring

Two questions in the dynamic protocol will assess appetite and food intake. Food ingestion will also be assessed and paired with activity monitoring using the arm band data.

#### Emotional state during EMA process

The wearable tracker band will record electrodermal activity at 3 time points: 5 min prior to EMA questioning; during the patient answer to EMA and 5 min after. Objective longitudinal records from patients will determine the sleep-suicide behavior casual chain based on wearables and EMA records.

### Sample size

We propose to recruit 1000 patients from two sites and expect a total of 100 suicide re-attempts during the follow-up period. We plan to recruit 500 patients per site (total of 1000) during 18 months with a follow-up period of 6 months. Based on our suicidal behavior monitoring pilot data (see above), we have calculated power in multivariate logit regression models, assuming a type I error of 0.05 and two-sided comparison. Using the effect sizes and standard deviations from our pilot data we expect our sample 1000 attempters and 100 reattempters during the follow-up) to provide 80% power to detect significant changes in death desire, suicidal ideation or suicide attempt related with sleep and appetite.

### Data analysis

We will carry out descriptive analyses of all data collected separately by site and treatment. We will calculate means, variances and empirical quartiles, and assess the distribution and skewness of the data. If groups differ significantly on covariates then these scores will additionally be included as covariates in multivariate regression models.

We will use 3 multi-level logit regression to account for multiple observations per individual, to identify individual-level (sleep, appetite, socio-demographic, clinical data, treatment data) and site-level characteristics associated with death desire, suicidal ideation or suicide attempt. We will use hazards models to relate covariate characteristics (sleep, appetite, socio-demographic, clinical data, treatment data) with time to suicide re-attempt during the follow-up period**.** Data mining (machine learning) techniques will be used to examine risk factors, patterns of illness evolution and outcomes, and patient stratification by level of risk.

## Discussion

### Data privacy

Data privacy is a serious concern in the e-health research area. Our system complies with the highest level of security regarding patients’ data. The eB2 app captures data from smartphones, which possibly was a deterrent for patients to accept the app [40]. In a preliminary study, patients were aware of the general approach of our method and were not very concerned about sharing their personal data since it was anonymized at the smartphone. Another major concern regarding personal electronic data is data security [41]. In order to preserve the patient’s privacy and reduce the risk associated to a non-legitimate access, all the sensible information stored in the eB2 server was hashed and anonymized. Phone numbers, email addresses, Bluetooth, and Wi-Fi MAC addresses were hashed using the SHA-1 algorithm and the location was transformed using a non-invertible function. We store randomly-rotated relative location coordinates, where the origin was the location that was most common during the first three days after installation (typically the patient’s home). Our apps are available via app stores, which allows us to continuously update and improve the app based on newly discovered bugs and also user feedback. However, the access to the apps contents and features is permitted only for patients accepting to participate to the study. All online self-report data are de-identified (identified by a random code, unrelated to any data protected by HIPAA). Only the PI holds the code, stored in a secure location separate from data. Personal information about potential and enrolled participants will be collected, and maintained in order to protect confidentiality before, during, and after the trial according to the terms described in the ethic committee report. These data will can be used for anciliary analysis after the study manuscript publication. Data will only be shared by the cited authors of this manuscript. Data will be anonymized before communication to other research office members .

### The burden of EMA method

Other important ethical are related to EMA method must also be considered. The matter of participant burden is one such issue. EMA methods entails a non-negligible time commitment on behalf of the participants. EMA rely on daily, or sometimes more frequent, Smartphone notifications. Notifications produce a significant increment in verified compliance when compared with an identical trial without notification. A significant eroding of compliance can be observed after 3 weeks of ecological assessment [42]. Furthermore, recording participants’ daily experiences in a continuous manner is an integral part of EMA. This approach may be significantly more invasive than asking a participant to complete a retrospective questionnaire or answering a question at a traditional interview [43]. The risk of intrusiveness into daily life is real. Receiving notifications may also inconvenience participants who are expected to complete an EMA or read information at a moment’s notice (ie, requests may occur at inopportune times).

### A paradigm shift in suicide risk prevention

This study stands out from randomized controlled trials or observational studies ailing to identify suicide risk factors.

We conducted a preliminary study showing that the eB2 system was capable of identifying mobility pattern changes, which may be used as proxies for behavioral changes and relapses in mental health [6]. We will apply the two key type of models previously tested in suicidal prediction: 1) predictive models or discriminative models and 2) explanatory or generative models. Both predictive and explanatory models use patient features to provide information on future events, such as the likelihood that a patient will attempt suicide in a given time interval. Applying these models to our database, will generation of tools to improve medical decision-making, patient outcomes, and to identify mediators and moderators within causal pathways. We will also develop latent feature models using Nonparametric Bayesian (NPB) approaches to detect the different predictive signatures. The central idea behind nonparametric Bayes (NPB) is the replacement of the classical finite-dimensional prior distributions with general stochastic processes, allowing an open-ended number of degrees of freedom in a model. Nonparametric priors have several properties that make them desirable to model data with an unknown number of characteristics of different sizes or importance.

Overall, we believe that “Smartcrises” could participate to a paradigm shift from the traditional identification of risks factors in mental illnesses to personalized prevention strategies tailored to characteristics for each patient, i.e., personalized medicine [44]. We have developed a system that is able to capture data from the smartphone’s native sensors and other wearables. AI techniques seek to answer the question: How can we build computer systems that automatically improve with experience, and what are the fundamental laws that govern all learning processes? Machine learning techniques allow processing of real-time observational information by continuously learning from data to build understanding and uncover previously unexpected associations and patterns in data. Predictive and explanatory models might use individual patient data to predict future events like the probability of a patient attempting suicide in a given time interval. Our final goal would be the identification of more general behavioral changes (online social interaction) in outpatients, which has important applications for a wide range of chronic conditions, including mental health disorders. Apart from the continuous assessment of bio-parameters themselves, smartphone-based monitoring would also allow researchers to gather information on context and environment, which may prove valuable for the interpretation of the monitored biomedical data and allow for a better preventative approaches.

## Data Availability

All datasets on which the conclusions of the paper rely should be available to readers except the source code of the smartphone apps developed for the study.

## Abbreviations

AI: Artificial intelligence
EMA: Ecological momentary assessment
ESM: Experience sampling methods
